# Systemic Alterations of Immune Response-Related Proteins during Glaucoma Development in the Murine Model DBA/2J

**DOI:** 10.3390/diagnostics10060425

**Published:** 2020-06-23

**Authors:** Andrés Fernández-Vega Cueto, Lydia Álvarez, Montserrat García, Enol Artime, Ana Álvarez Barrios, Ignacio Rodríguez-Uña, Miguel Coca-Prados, Héctor González-Iglesias

**Affiliations:** 1 Instituto Oftalmológico Fernández-Vega, Avenida Doctores Fernández-Vega, 34, 33012 Oviedo, Spain; afvega89@gmail.com (A.F.-V.C.); mgarciadiaz@fio.as (M.G.); irodriguezu@fernandez-vega.com (I.R.-U.); 2 Instituto Universitario Fernández-Vega (Fundación de Investigación Oftalmológica, Universidad de Oviedo), 33012 Oviedo, Spain; enol.artime@fio.as (E.A.); uo245562@uniovi.es (A.Á.B.); miguel.coca-prados@yale.edu (M.C.-P.); 3 Department of Ophthalmology and Visual Science, Yale University School of Medicine, New Haven, CT 06510, USA

**Keywords:** glaucoma, DBA/2J mouse model, blood, biomarkers, immune response, complement system

## Abstract

Animal models of glaucoma, a neurodegenerative disease affecting the retina, offer the opportunity to study candidate molecular biomarkers throughout the disease. In this work, the DBA/2J glaucomatous mouse has been used to study the systemic levels of several proteins previously identified as potential biomarkers of glaucoma, along the pre- to post-glaucomatous transition. Serum samples obtained from glaucomatous and control mice at 4, 10, and 14 months, were classified into different experimental groups according to the optic nerve damage at 14 months old. Quantifications of ten serum proteins were carried out by enzyme immunoassays. Changes in the levels of some of these proteins in the transition to glaucomatous stages were identified, highlighting the significative decrease in the concentration of complement C4a protein. Moreover, the five-protein panel consisting of complement C4a, complement factor H, ficolin-3, apolipoprotein A4, and transthyretin predicted the transition to glaucoma in 78% of cases, and to the advanced disease in 89%. Our data, although still preliminary, suggest that disease development in DBA/2J mice is associated with important molecular changes in immune response and complement system proteins and demonstrate the utility of this model in identifying, at systemic level, potential markers for the diagnosis of glaucoma.

## 1. Introduction

Glaucoma is a complex group of neurodegenerative disorders characterized by the progressive degeneration of the optic nerve, retinal ganglion cell (RGC) death and the loss of visual field [1]. Global glaucoma prevalence is currently estimated in 80 million people, while the projected number of affected will increase to 118 million in 2040 [2,3]. Primary open-angle glaucoma (POAG) and pseudoexfoliation glaucoma (PEXG) are the most prevalent types of glaucoma in developed countries [4,5]. Both subtypes of glaucoma are multifactorial in origin, sharing an abnormal increase in the intraocular pressure (IOP) related with their onset. The IOP elevation is associated with a dysfunction of the normal flow of the aqueous humor, either because of an excessive production of this fluid or due to the obstruction of the outflow system triggered by an accumulation of aggregates in the trabecular meshwork [6,7].

It is often clinically observed that by the time a glaucoma patient is diagnosed, he/she has already lost 35–40% of his/her RGCs [8]. Therefore, there is a current need for a much more sensitive and specific method of early detection of glaucoma for an effective treatment and the improvement of its prognosis. Molecular biomarkers of glaucoma are potentially beneficial in the early diagnosis and management of this eye disease, leading to a better understanding of its pathophysiology [9]. Studies aiming the discovery of molecular biomarkers for potential clinical application have been focused in the analysis of tears, aqueous and vitreous humor, and blood/serum [10,11,12,13]. In a previous study, we identified alterations in serum proteins when comparing patients with POAG, PEXG and healthy controls by differential proteomics analysis [14]. In that seminal work, a panel of the top-17-ranked distinct proteins, the signaling network of which correlated to immunological and inflammatory response pathways, was confirmed by immunoassays (ELISA), with apolipoprotein A4 (APOA4) classifying the groups with 81% of correct assignment.

Considering the challenge to follow-up any protein along the pre- to post-glaucomatous transition in humans, the use of animal models of glaucoma may offer an opportunity to verify the applicability of the identified proteins as candidate biomarkers and/or their therapeutic potential to prevent/block the progression of the disease. The mouse strain DBA/2J is widely used since it manifests many of the pathological features of human glaucoma, including IOP elevation, progressive damage to the optic nerve and loss of RGCs [15,16]. The DBA/2J mice present two mutations in the *Gpnmb* and *Tyrp* genes, producing iris pigment dispersion (IPD) and iris stromal atrophy (ISA), resulting in age-related elevation of IOP and the consequent development of glaucomatous pathology in approximately 70% of these mice [17,18,19]. At 4 months DBA/2J mice did not present any sign of glaucomatous disease, including IOP elevation, significant loss of axons in the optic nerve, or changes in the anterior chamber such as ISA and IPD. At 10 months old, the IOP reaches maximum levels and severe loss of axons is observed in 34% of the animals indicating the onset of glaucoma. Finally, at 14 months the DBA/2J mice developing glaucoma show advanced features of the disease [16]. As genetically matched control strain, the D2G mice have the same genetic background as DBA/2J but with the functional allele of *Gpnmb*, so they do not develop elevated IOP or glaucoma, although they exhibit a mild ISA [20].

In this preliminary study we aim to evaluate the usefulness of the glaucoma animal model DBA/2J in the search for potential systemic biomarkers of the disease. To this end, the DBA/2J mice have been used to determine the serum alterations of several candidate glaucoma biomarkers previously discovered in humans, throughout the stages of the disease. We examined their potential systemic alterations in the transitions between the pre- and glaucomatous phases, i.e., between 4 and 10 months, and in the final glaucomatous stage at 14 months. In this study, ten proteins were selected from the 17-top-ranked altered previously identified in humans, based mainly on their highest classification power among glaucoma and healthy control groups, by means of Naïve Bayes algorithm and protein classification for predicting glaucoma, considering correct assignment, sensitivity, specificity and area under the curve [14]. Quantitation of selected proteins, related to the immune response and the complement system, included: APOA4, complement C3 (C3), transthyretin (TTR), complement C4a (C4a), inter-alpha-trypsin inhibitor heavy chain H4 (ITIH4), apolipoprotein A1 (APOA1), alpha 1-antitrypsin (a1AT), complement factor H (CFH), apolipoprotein L1 (APOL1), and ficolin-3 (FCN3). Systemic variations of immune response-related and complement system proteins were identified in the serum of DBA/2J mice, along the progression of the glaucomatous disease up to the onset of the pathophysiological manifestations. As a proof of concept, the use of a five-protein panel, consisting of C4a, CFH, FCN3, APOA4, and TTR predicts the transition to glaucoma disease in 78% of cases.

## 2. Materials and Methods

### 2.1. Mouse Strains, Breeding, and Husbandry

DBA/2J and DBA/2J-*Gpnmb* ^+^/SjJ (D2G) mice of different sex and age were used. D2G (JAX stock #007048) and DBA/2J (JAX stock #000671) mice were initially obtained from The Jackson Laboratory (Bar Harbor, ME, USA) and the European distributor of Jackson Laboratories Mice (Charles Rivers Laboratories, Wilmington, MA, USA), respectively.

Mice were maintained on a 12-h light–dark cycle, where humidity and temperature were controlled, with food and water available ad libitum. All experimental procedures followed the ARRIVE (Animal Research: Reporting of In Vivo Experiments) guidelines [21], and were carried out in accordance with the European Communities Council Directive (2010/63/UE) on the protection of animals used for scientific purposes and the Spanish regulations on the protection of animals used for research. The study was approved by the Animal Research Ethics Committee (Code 11-INV_2013, approved on 20 December 2013) of the Principality of Asturias (Oviedo, Spain).

### 2.2. Experimental Design

**Figure 1:**
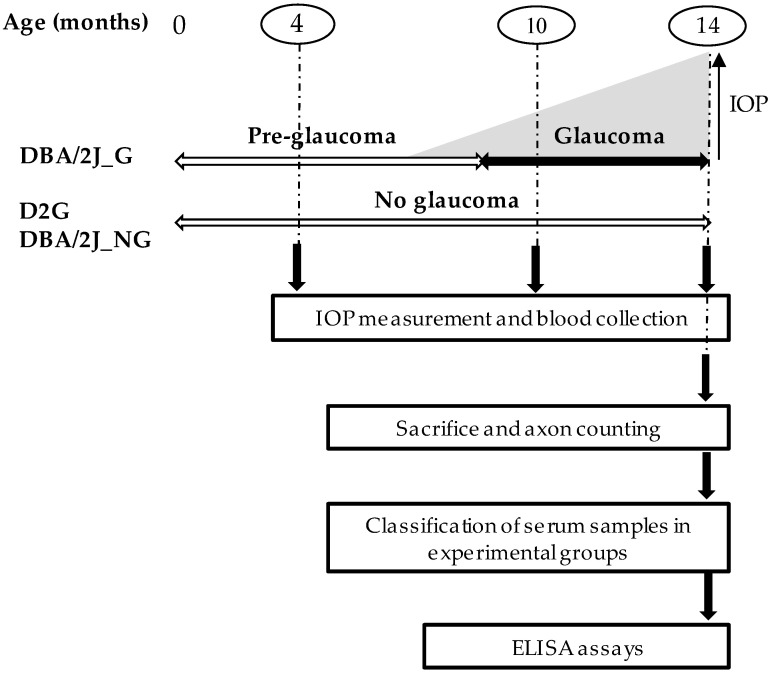
Experimental design. Intraocular pressure (IOP) analysis and blood collection were carried out at 4, 10, and 14 months for systemic proteins screening. The D2G and DBA/2J mice were ultimately sacrificed at 14th month and serum samples classified according to the number of axons. DBA/2J_G: mice that developed glaucoma with the age; D2G and DBA/2J_NG: mice without signs of glaucoma.

The experimental design is shown in Figure 1. The IOP was measured and blood was drawn from all mice (D2G and DBA/2J) at 4, 10, and 14 months of age. After 14 months, the mice were sacrificed by cervical dislocation and the optic nerves were processed for the subsequent axonal damage assessment. The serum samples were classified into several experimental groups based on the estimated pathological state of the mouse at the time of blood sampling. Glaucomatous state was inferred according to the optic nerve damage at the time of the sacrifice. The DBA/2J mice that did not develop glaucoma (DBA/2J_NG) were used as controls to evaluate any possible existing age-related changes in the levels of the studied molecular markers. However, considering that the expected number of DBA/2J_NG mice would be 30% maximum, and in view of the number and amount of these control samples not being sufficient for the analysis of all the proposed proteins, the D2G strain was included as an additional control group to verify existing age-related differences in the remainder studied proteins.

### 2.3. Intraocular Pressure (IOP) Measurement

A Tonolab^®^ rebound tonometer (Icare LAB tonometer, TonoLab, Vantaa, Finland) was used to measure the IOP of D2G and DBA/2J mice [22,23]. This portable impact tonometer is specifically designed for its use in rodents (rat and mouse) [24]. IOP measurements were performed in both eyes of all mice of our colonies at 4, 10, and 14 months old. Additional measurements were taken in some mice, randomly selected, of both strains at 2, 6, and 8 months old in order to determine more accurately the evolution of IOP with age. IOP measurements from eyes showing corneal calcification were discarded. All IOP analyses were carried out by the same examiner, according to the procedures recommended by the manufacturer. To prevent instrument movements during the measurement procedure and to maintain the horizontal orientation of the probe, the tonometer was fixed in a vertical position to a support stand by means of a clamp. Each animal was individually placed on a platform, in which the optical axis of the analyzed eye was aligned with the probe tip, at a distance of 2–3 mm approximately.

Mice were anesthetized with an intraperitoneal injection of a solution of ketamine (50 mg/kg, Ketamidor, Ritcher Pharma, Wels, Austria) and xylazine (10 mg/kg, Rompun, Bayer AG, Leverkusen, Germany) and body temperature was maintained using a heating pad. The potential effects of anesthesia on IOP record were controlled by immediately measuring the IOP once the mouse failed to respond to touch, typically within 2 to 5 min of loss of consciousness [25]. To control for the diurnal variation in IOP [25,26,27], all measurements were taken at the same time in the morning, starting at 10 am. The Tonolab^®^ performs 6 instrumental readings per measurement. Moreover, to obtain reliable data, five consecutive measurements from each eye were performed, obtaining the average for the left and right eyes, respectively.

### 2.4. Blood Sampling Collection

Blood was drawn from conscious mice through submandibular vein puncture at 4, 10, and 14 months of age. Then 25 to 150 μL of blood was collected from each mouse at all selected ages. Samples were allowed to clot for 1 h at 37 °C and 6 h at 4 °C, and then centrifuged at 2000 rpm for 5 min at 4 °C. The supernatants, i.e., serum, were collected and stored at −80 °C until analysis.

### 2.5. Optic Nerves Dissection, Fixation, and Staining

Once the mice were sacrificed, they were decapitated and each whole head was fixed in 1% paraformaldehyde, (Sigma-Aldrich, St. Louis, MO, USA), 1% glutaraldehyde (Sigma-Aldrich, St. Louis, MO, USA), 0.2 M phosphate buffer (Sigma-Aldrich, St. Louis, MO, USA), pH 7.4, overnight at 4 °C. Then, eyes with approximately 3 mm of intracranial portions of optic nerves were carefully dissected and preserved in the fixative overnight at 4 °C. Subsequently, eyes with the optic nerves attached were rinsed twice for 15 min in 0.1 M phosphate buffer at 4 °C, and the most distal part of the optic nerve to the eyeball was knotted with a piece of suture thread to distinguish the regions proximal and distal to the eye cup. Then, the optic nerves were separated from the globe and processed as previously described [16,18,28,29,30]. In brief, dissected optic nerves were fixed in 1% osmium tetroxide (Sigma-Aldrich, St. Louis, MO, USA) for 4 h at 4°C. After osmification, nerves were rinsed three times for 10 min in 0.1 M sodium-acetate buffer pH 6.0 and dehydrated in graded ethanol concentrations. Finally, they were embedded in a mixture of Araldite and Epon resins (Araldite 502/PolyBed 812 kit, Polysciences Inc., Heidelberg, Germany). Semi-thin sections (1 µM) of the anterior segment of the nerves (proximal to the eyeball) were perpendicular cut to their long axis and stained with 1% paraphenylenediamine (PPD, Sigma-Aldrich, St. Louis, MO, USA) for 30 min. PPD darkly stains the myelin sheaths and axoplasm of injured or dying axons, but not healthy axons, allowing the detection of a single damaged axon within the optic nerve [28,31].

### 2.6. Optic Nerve Damage and Mice Stratification

Two semi-thin sections (1 µm thick) of each nerve were selected based on their contrast and the number of axons was determined. Axon counting allowed the identification of DBA/2J mice that did not develop glaucoma (DBA/2J_NG) and glaucomatous DBA/2J (DBA/2J_G). It must be stressed that not all glaucomatous mice developed the disease in both eyes. To obtain the area of each selected optic nerve cross section, a 10x magnification photograph was taken in which the optic nerve was manually outlined and its area was automatically calculated with the software ImageJ [32]. The average of three independent measures was obtained for each section.

The optic nerve damage was evaluated through established unbiased counting methods. To this end, a microscope LEICA CTR 6000, equipped with an automated motorized-stage, a digital camera LEICA DFC310 FX, and the program LAS X (Leica Application Suite X, Leica Microsystems, Wetzlar, Germany) for image processing was used. The four cardinal points of each selected section were marked in the optic nerve image (100× magnification), delimiting a square or rectangle including the complete optic nerve cross section. A series of 20–40 photomicrographs, 6000 µm^2^ area each, with none or minimal overlapping was taken automatically, which represents the entire section of the optic nerve. A rectangle of 1000 µm^2^ was drawn exactly in the center of each photo with the program Adobe Photoshop CS6 (Adobe Inc., San Jose, CA, USA) and the axons included into were marked and manually counted using the program ImageJ software (National Institute of Health, MD, USA). The central area of each photo was selected to avoid field overlapping, and therefore the same area was not counted twice. The photo was discarded when the central rectangle included an area >30% outside the optic nerve section. Conversely, when the central rectangle of a photo included an area <30% outside the optic nerve section, the delimited area of the optic nerve was measured and its axons counted. The average area counted for each optic nerve section was in the range from 10.2% to 20.1% of the total area. The total number of axons in the entire area of the optic nerve of each eye was extrapolated from the number of axons in the counted area. The average number of axons in the 2 sections counted was obtained for each optic nerve, and the final value was expressed as number of axons per optic nerve.

Optic nerve damage level was graded into three stages [18,20,30,33,34,35]: (i) Nerves with none or early damage (NOE), without detectable axon damage compared with non-glaucomatous controls (less than 5% axons damaged). (ii) Nerves with moderate damage (MOD) presented between 10–50% of axon loss, but with mostly healthy remaining axons. This stage was rarely observed in non-glaucomatous mice. (iii) Nerves with severe glaucoma (SEV), presenting extensive axon damage with at least 50% axonal loss.

### 2.7. Experimental Groups

**Figure 2:**
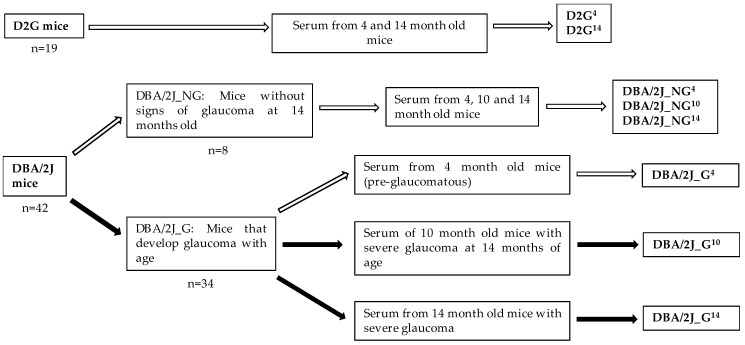
Experimental groups. Control groups: D2G^4^, D2G^14^, DBA/2J_NG^4^, DBA/2J_NG^10^, and DBA/2J_NG^14^. Glaucoma groups: DBA/2J_G^4^, DBA/2J_G^10^, DBA/2J_G^14^. White arrows indicate samples of non-glaucomatous mice. Black arrows indicate samples of glaucomatous mice.

Mice were distributed in three experimental groups (Figure 2) according to the axon counting after the sacrifice of the animals at 14 months of age: D2G strain (D2G), DBA/2J that did not develop glaucoma throughout their life (DBA/2J_NG) and glaucomatous DBA/2J (DBA/2J_G). Mice serum samples obtained at different ages were classified in the following subgroups: (i) serum samples from D2G mice at 4 and 14 months i.e., D2G^4^ and D2G^14^, respectively; (ii) serum samples from 4, 10, and 14 months old DBA/2J_NG mice, i.e., DBA/2J_NG^4^, DBA/2J_NG^10^, DBA/2J_NG^14^, respectively; (iii) serum samples from 4 months old DBA/2J_G mice, in pre-glaucomatous stage, that developed glaucoma with age, i.e., DBA/2J_G^4^, serum samples from 10 months old DBA/2J_G mice with severe glaucoma at 14 months in at least one eye and unknown glaucomatous stage at 10 months, i.e., DBA/2J_G^10^, and serum samples from 14 months old DBA/2J_G mice with severe glaucoma in at least one eye, i.e., DBA/2J_G^14^. Serum samples from 10 months old D2G (D2G^10^) were not used in posterior analysis and are therefore not included in the experimental groups.

### 2.8. ELISA Assays

Quantitation of 10 proteins of interest in serum samples was performed using commercially available ELISA assays (SEB967Mu for APOA4, SEA861Mu for C3, SEA726Mu for TTR, SEA389Mu for C4a, SEH776Mu for ITIH4, SEA519Mu for APOA1, SEB697Mu for a1AT, SEA635Mu for CFH, SED741Mu for APOL1, and SEB903Mu for FCN3), following the instructions described by the manufacturer (Cloud-Clone Corp., Houston, TX, USA). Concentrations of specific proteins were expressed in nanograms of the protein per microliter of serum. Representative analytical parameters for each of ELISA assays have been included in Supplementary Table S1.

### 2.9. Statistical Analysis

The five measurements of IOP in each eye were averaged and the mean was calculated with the values of both eyes to give a single IOP value per animal. All data were reported as mean ± standard error of the mean. Comparisons between IOP values obtained at different ages in both strains were carried out by non-parametric Mann–Whitney test, using GraphPad Prism version 3 for Windows (GraphPad Software, San Diego, CA, USA).

Non-parametric Kruskal-Wallis test (Dunn’s test for Multiple Comparisons) with GraphPad Prism version 3 for Windows was used to analyze differences in concentration of serum proteins between the groups. A *p*-value less than 0.05 was considered statistically significant. Further stepwise discrimination analysis was carried out with SPSS version 15.0 (IBM Corp., Armonk, NY, USA) using the concentrations of the proteins determined by ELISA in the different sample groups. Subsequently, several statistical tools based on Machine Learning approaches were applied using Orange Canvas software v2.6 (http://orange.biolab.si) to assess which method provided the best accuracy for the correct classification of samples based on the selected panel of protein markers. These tools included Receiver Operating Characteristic curve analyses for each of the markers, Naive Bayes, k-Nearest Neighbor, Random Forest, Classification Trees, and Support Vector Machine [36].

Protein–protein interaction (PPI), significant pathways and networks associated to the ELISA-quantified proteins were predicted using the Search Tool for Retrieval of Interaction Genes/Proteins (STRING, version 11.0, https://string-db.org/) [37] database of physical and functional interactions. Network analysis was set at medium stringency (STRING score = 0.4) and proteins were linked based on seven interaction sources: experiments, databases, text mining, co-expression, neighborhood, gene fusion, and co-occurrence.

## 3. Results

### 3.1. Animals, Optic Nerve Axons Number, and IOP

**Figure 3:**
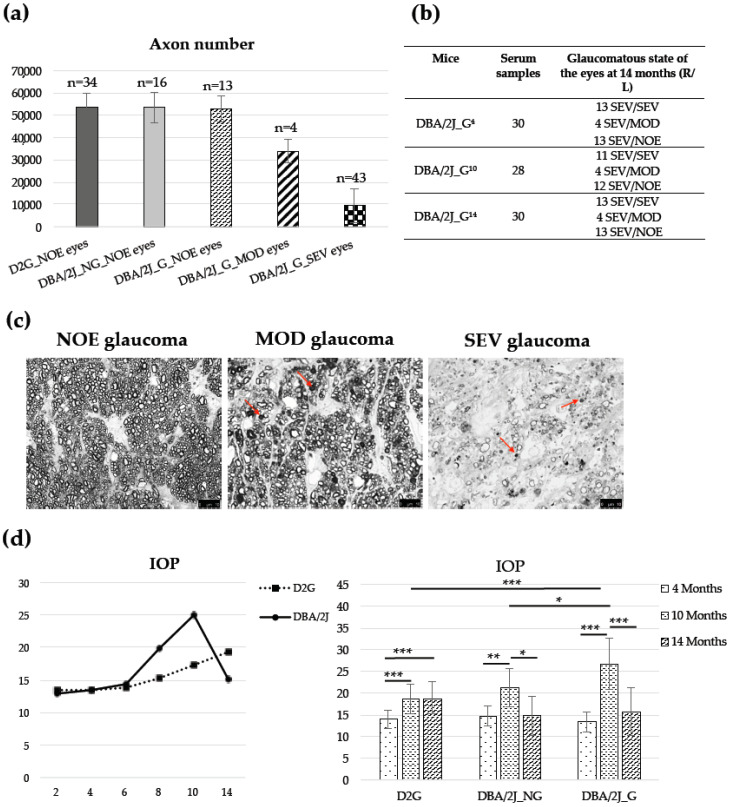
(**a**) Axon number in the optic nerves of D2G and DBA/2J mice at 14 months old. NOE, no or early glaucoma; MOD, moderate glaucoma; and SEV, severe glaucoma. The average number of axons and the standard deviation are represented (n, number of eyes). (**b**) Serum samples from DBA/2J_G mice at 4, 10 and 14 months and glaucomatous state of the eyes of these mice at 14 months. (**c**) Representative images of optic nerve damage levels in DBA/2J mice 14 months old: NOE glaucoma, few or no damaged axons in the optic nerve; moderate glaucoma, clear presence of damaged axons and axon loss, and severe glaucoma, massive axon loss. Arrows show damaged axons that stain darkly with PPD. Images were taken from semi-thin sections of the retro-orbital region of the optic nerve stained with PPD Scale bar 10 µM. (**d**) IOP evolution with age. Left, IOP evolution in the D2G and DBA/2J colonies. Right, IOP values in the mice D2G, DBA/2J_NG, and DBA/2J_G used for serum extraction at 4, 10, and 14 months old. *, *p*-value < 0.05; **, *p*-value < 0.01; ***, *p*-value < 0.001.

Figure 3 (panel a) summarizes the average of axons of the eyes of 38 DBA/2J (24 females and 14 males) and 17 D2G (13 females and 4 males) mice of 14 months of age. As expected, none of the D2G mice showed any signs of glaucoma in their optic nerves. Among the DBA/2J strain, 8 mice (6 females and 2 males) did not develop glaucoma (i.e., 21%, DBA/2J_NG), while the rest (30 mice, 18 females, and 12 males) developed glaucoma (DBA/2J_G), all of them severe in at least one eye at 14 months old (13 SEV/SEV, 4 SEV/MOD and 13 SEV/NOE). The average axons of optic nerves classified as NOE were 53,449 ± 6335 (34 eyes), 53,412 ± 6985 (16 eyes), and 52,614 ± 5840 (13 eyes) for D2G, DBA/2J_NG, and DBA/2J_G, respectively, without significant differences between groups (*p*-value > 0.05). The average axons of optic nerves of DBA/2J_G with MOD glaucoma was 33,816 ± 5361 (4 eyes) and for optic nerves with SEV glaucoma was 9456 ± 7813 (43 eyes), with significant differences between SEV and MOD optic nerves, *p*-value < 0.01. Similarly, significant differences were found when comparing SEV or MOD optic nerves of DBA/2J_G with NOE optic nerves of D2G or DBA/2J mice, *p*-values < 0.001.

The number of serum samples from DBA/2J_G mice at 4, 10, and 14 months and the glaucomatous state of the eyes of these mice at 14 months are shown in panel b of Figure 3. Same Figure 3 (panel c) shows the representative images of optic nerve damage levels in 14 months old DBA/2J mice: NOE, with no or early glaucoma; MOD, with clear presence of damaged axons and axon loss, and SEV, with massive axon loss.

IOP measurements are depicted in panel d of Figure 3. To follow the evolution of intraocular pressure with age, the IOP values were obtained at 4, 10, and 14 months in all D2G and DBA/2J mice of our colonies (32 D2G, 18 females, and 14 males; 59 DBA/2J, 39 females, and 20 males), and at 2, 6, and 8 months old in an additional subset of mice randomly selected of both strains (see panel d, left chart). It should be noted that the DBA/2J colony includes both mice developing (DBA/2J_G) and not developing glaucoma (DBA/2J_NG). According to the figure, the IOP of the DBA/2J strain raised after 6 months of age until 10 months, while at 14 months recovered normal values, while the IOP of the D2G mice slightly increased with age in agreement with the literature [15]. No influence of sex over IOP was observed in our DBA/2J and D2G colonies, p-value>0.05. Similarly, the IOP data of the D2G and DBA/2J mice used for serum extraction and protein quantitation at 4, 10, and 14 months (17 D2G, 13 females, and 4 males; 8 DBA/2J_NG, 6 females and 2 males; 30 DBA/2J_G, 18 females and 12 males) are also plotted (panel D, right chart) where the IOP of DBA/2J_NG and DBA/2J_G mice are separately represented. As can be observed, the increase of IOP at 10 months of age compared to 4 months was significantly more pronounced in the DBA/2J_G mice, *p*-value < 0.001, than in the control group DBA/2J_NG, *p*-value < 0.01.

### 3.2. Quantification of Serum Proteins by ELISA Analysis

The concentrations (ng protein/µL serum, expressed as mean value and standard deviation) for each of the proteins estimated by ELISA in DBA/2J_G^4^, DBA/2J_G^10^, DBA/2J_G^14^, and in DBA/2J_NG^4^, DBA/2J_NG^10^, DBA/2J_NG^14^ or in D2G^4^ and D2G^14^ groups, are shown in Table 1. To observe any age-related changes in the studied proteins, the DBA/2J_NG was used as control group for half of the analyzed proteins, while the D2G group was used as alternative control for the remaining proteins, since the number of DBA/2J_NG mice was limited and therefore there were not enough serum samples to perform all analyses.

The Kruskal-Wallis test (non-parametric ANOVA) was used to compare the concentration of each of the proteins between groups. Based on this test, in five of the ten proteins individually analyzed, i.e., APOA1, C3, a1AT, APOL1, and FCN3, no significant differences were observed among the glaucoma sample groups (DBA/2J_G^4^ vs. DBA/2J_G^10^ and DBA/2J_G^14^, i.e., preglaucomatous mice at 4 months and glaucomatous mice at 10 and 14 months old). Similarly, the ten analyzed proteins did not show age-related changes (D2G^4^ vs. D2G^14^, *p*-value > 0.05; DBA/2J_NG^4^ vs. DBA/2J_NG^14^, *p*-value > 0.05).

**Figure 4:**
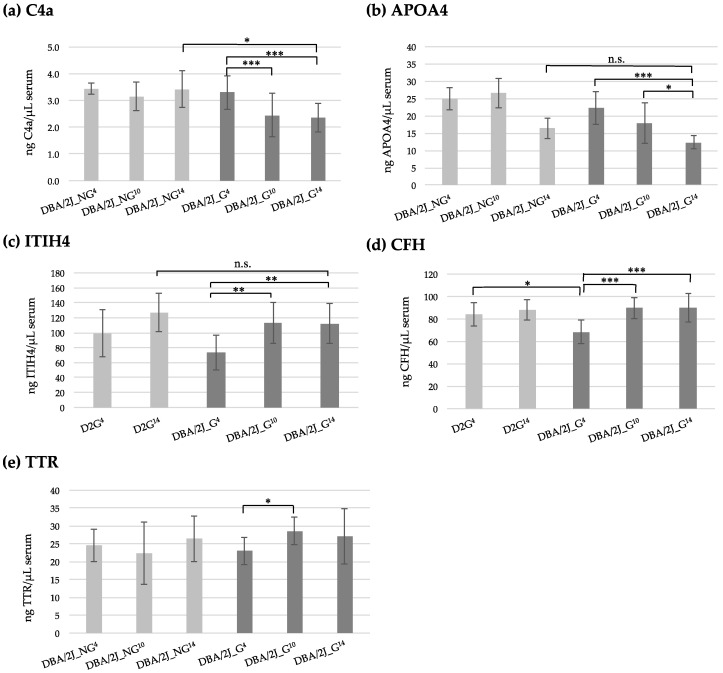
(**a**) Concentration of protein C4a (ng of protein per μL of serum) in DBA/2J_NG^4,10,14^ and DBA/2J_G^4,10,14^ groups. (**b**) Concentration of protein APOA4 (ng of protein per μL of serum) in DBA/2J_NG^4,10,14^ and DBA/2J_G^4,10,14^ groups. (**c**) Concentration of protein ITIH4 (ng of protein per μL of serum) in D2G^4,14^ and DBA/2J_G^4,10,14^ groups. (**d**) Concentration of protein CFH (ng of protein per μL of serum) in D2G^4,14^ and DBA/2J_G^4,10,14^ groups (**e**) Concentration of protein TTR (ng of protein per μL of serum) in DBA/2J_NG^4,10,14^ and DBA/2J_G^4,10,14^ groups. The *p*-values were obtained by the Kruskal-Wallis test with Dunn’s Multiple Comparison test. n.s., not significant; *, *p*-value < 0.05; **, *p*-value < 0.01; ***, *p*-value < 0.001.

As shown Figure 4, the proteins C4a (panel a) and APOA4 (panel b) were found in lower concentrations in DBA/2J_G^10^ and DBA/2J_G^14^ groups when compared to DBA/2J_G^4^ group, depicting a age- and/or disease-related decrease in the DBA/2J mice developing glaucoma. Regarding C4a, lower levels were found in DBA/2J_G^10^ and DBA/2J_G^14^ mice when compared with preglaucomatous DBA/2J_G^4^ mice (0.74- and 0.71-fold, respectively, *p*-values < 0.001). Additional significant differences were observed for this protein when compared DBA/2J control mice at 14 months (DBA/2J_NG^14^) with DBA/2J mice with severe glaucoma mice at 14 months (DBA/2J_G^14^) (0.68-fold, *p*-value < 0.05). Conversely, the C4a concentration in the DBA/2J_NG control group remained constant at 4 and 14 months (*p*-value > 0.05). On the other hand, the levels of APOA4 significantly decreased in DBA/2J_G^14^ group when compared to DBA/2J_G^4^ group (0.56-fold, *p*-value < 0.001). Surprisingly, these changes are not due to age-related variations, since APOA4 levels were not significantly different in DBA/2J_NG groups at 4 and 14 months (*p*-value > 0.05, see Table 1).

The proteins ITIH4 and CFH (panels c and d of Figure 4) were found in higher concentrations in 10 and 14 month old glaucomatous mice (DBA/2J_G^10^ and DBA/2J_G^14^) compared to preglaucomatous stage (DBA/2J_G^4^), therefore suggesting changes in the transition phase to glaucoma. Specifically, the concentration of ITIH4 significantly increased in DBA/2J_G^10^ and DBA/2J_G^14^ groups when compared with DBA/2J_G^4^ (1.5-fold, *p*-values < 0.01), while no significant differences were found between control D2G mice at 4 and 14 months old. Likewise, the CFH protein concentration was significantly elevated (1.3-fold DBA/2J_G^10^ and DBA/2J_G^14^ groups) when compared to DBA/2J_G^4^ (*p*-value < 0.001), but no differences were found between D2G controls (4 vs. 14 months, *p*-value > 0.05). Interestingly, the DBA/2J that will develop glaucoma presented significantly lower levels of CFH at 4 months than the control strain at the same age (DBA/2J_G^4^ vs. D2G^4^, 0.81-fold change, *p*-value < 0.05).

Finally, the TTR protein (panel e of Figure 4) was significantly over-expressed in DBA/2J_G^10^ group (1.25-fold change, *p*-value < 0.05), when compared to DBA/2J_G^4^ control mice. However, no significant differences were found when comparing 4 months mice that will develop glaucoma (DBA/2J_G^4^) with 14 months glaucomatous DBA/2J (DBA/2J_G^14^), like no differences were observed when comparing with DBA/2J that will not develop glaucoma (DBA/2J_NG^4 or14^).

### 3.3. Machine Learning Models

Based on the concentration found for each of the 10 proteins in serum samples from pre- and glaucomatous stages (DBA/2J_G^4^, DBA/2J_G^10^, and DBA/2J_G^14^), a stepwise discriminant analysis was performed to determine which variables (proteins) were the most important to differentiate between the groups. As a result of this feature-subset subtraction, five protein biomarkers appear to be the most significant in the discrimination of the studied groups. This panel included C4a, CFH, FCN3, APOA4, and TTR proteins, which were accordingly selected to generate multivariate predictive models. The usual strategy of machine learning is to split the data into training and evaluation subsets, using 70% of data to train models to see patterns and the 30% of data to evaluate the predictive quality of the trained model by comparing predictions on the evaluation data set with true values (stratified mice). To this end, we performed training using random sampling with 70% of the data and distinct machine learning algorithms, such as Naive Bayes, k-Nearest Neighbor, Random Forest, Classification Tree, or an ensemble of Support Vector Machine. Subsequently, the classification method was validated using the remaining 30% of data to make predictions on future cases for which the target answer is unknown.

As a result of this analysis, we found that the best classification efficiency was obtained when using the full panel of five markers (C4a, CFH, FCN3, APOA4, and TTR) and principally training the data with Naive Bayes and Random Forest, obtaining values of correct assignment of 62% when simultaneously comparing DBA/2J_G^4^, DBA/2J_G^10^, and DBA/2J_G^14^ groups, which percentage represents the correctly classified predictions. Interestingly, the five-protein panel correctly classified 89% of the mice when comparing DBA/2J_G^4^ vs. DBA/2J_G^14^ groups, 78% of mice when comparing DBA/2J_G^4^ vs. DBA/2J_G^10^ comparison, and 65% of mice when DBA/2J_G^10^ and DBA/2J_G^14^ groups were compared (see Table 2).

### 3.4. Functional Analysis and Protein–Protein Interaction (PPI) Network

**Figure 5:**
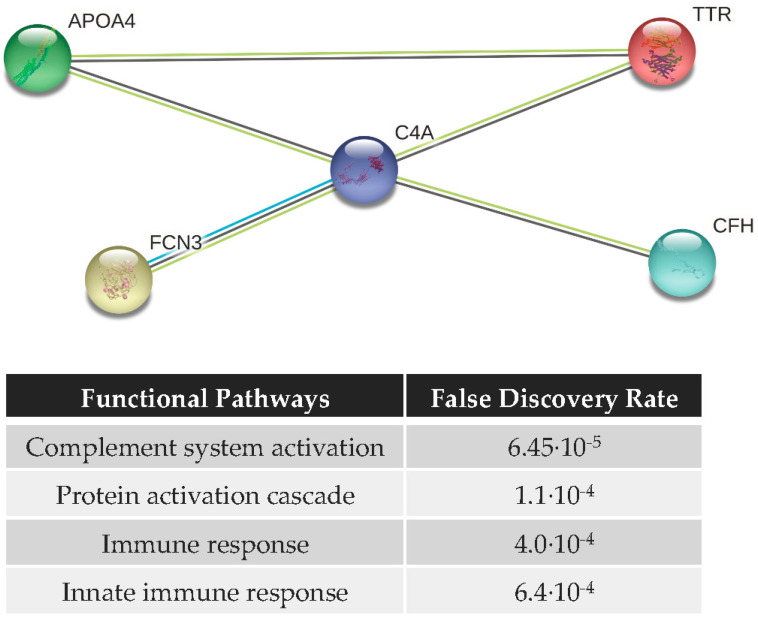
Protein–protein interaction (PPI) network of the most discriminating proteins (C4a, CFH, APOA4, TTR, and FCN3, after SPSS analysis) obtained by String software, containing 5 nodes (proteins) and 5 edges, with PPI enrichment *p*-value = 2.68 × 10^−7^.

Figure 5 shows the PPI network of the most discriminant 5-proteins (C4a, CFH, FCN3, APOA4, and TTR) analyzed by ELISA in mice, consisting of 5 nodes and 5 edges with PPI enrichment *p*-value < 2.68 × 10^−7^, which means that they are partially biologically connected as a group. Functional analysis indicates that the biological processes in which these proteins participate are related to the immune system. The statistically enriched pathways (*p*-value < 0.05) include: (i) complement system activation (*p*-value = 6.45 × 10^−5^); (ii) protein activation cascade (*p*-value = 1.1 × 10^−4^); (iii) immune response (*p*-value = 4.0 × 10^−4^); and (iv) innate immune response (*p*-value = 6.4 × 10^−4^), among others.

## 4. Discussion

The current study did not intend to discover the key molecules that mediate early damage in glaucoma, but rather to identify the potential of systemic levels of proteins as candidate biomarkers for an early diagnosis and/or better prognosis of the disease. To this end, as proof of concept the DBA/2J mouse model of glaucoma has been used considering its specific features of the human disease including age-related, elevation of IOP with variable onset, RGC loss, and progressive optic nerve axon damage [16,18]. Ten proteins previously identified as candidate biomarkers of human glaucoma were analyzed in the serum of DBA/2J mice at pre-glaucomatous (DBA/2J_G^4^) and glaucomatous stages (DBA/2J_G^10^ and DBA/2J_G^14^) of the later-onset glaucoma. To evaluate possible age-related changes that could confound the comparisons, serum samples from DBA/2J mice not developing glaucoma (DBA/2J_NG, 21% of the total number of DBA/2J mice) were used as controls for the analysis of five of the proteins (i.e., APOA1, APOA4, C3, C4a, and TTR). As an alternative, to compensate for the insufficient number and amount of samples obtained from DBA/2J_NG mice, the strain D2G was used as control for the analysis of the remainder proteins (i.e., a1AT, APOL1, CFH, ITIH4, and FCN3).

Dysregulation and activation of immune response and complement system are consistent features of glaucoma, both in humans and animal models [38,39]. The 10 serum-proteins analyzed along glaucoma development in DBA/2J mice belong to complement and coagulation systems and lipid metabolism networks, linked to immune and inflammatory-related processes [14]. Mutations in *Tyrp* and *Gpnmb* genes in DBA/2J result in the triggering of an immune response in the iris, leading to its atrophy, pigment dispersion and glaucoma [29]. There is strong evidence in early involvement of the immune system (immune response, stress response, and acute response) in the onset of glaucoma in DBA/2J, prior to the occurrence of the normal signs of glaucomatous disease [40]. An increase in the expression of genes related to the complement cascade, a major component of the innate immunity, has been previously described in the DBA/2J animal model, suggesting that early events in the pathology of glaucoma are associated with the activation of the complement system [33].

According to current literature, activation of the complement cascade occurs in both the retina and the optic nerve head in DBA/2J with glaucoma, but no study focused on the analysis of complement activation at systemic level. The protective or damaging role of complement system in DBA/2J mice is still under debate, acting as a double-edged sword depending on the disease stage [33,41], as well as the type of ocular disease [42]. As example, Bosco et al. (2018) recently uncovered a damaging effect of complement C3 or downstream complement activation in glaucoma [43], while Harder et al. (2017) proposed that C3 protects from early glaucomatous damage [35]. Interestingly, DBA/2J and D2G mice are naturally deficient in complement component 5 (C5), a key mediator of the downstream processes of the complement cascade, which may imply that upstream complement components are sufficient to drive neurodegeneration. On the other hand, C5 deficiency could protect DBA/2J mice from glaucoma since C5-sufficient DBA/2J mice develop a more severe glaucoma at an earlier age than standard DBA/2J mice [44].

As stated before, the significantly altered proteins in the pre- to glaucomatous transition stages of DBA/2J mice are related to immune and inflammatory processes. Complement system is an innate immune molecular network that acts to remove pathogens, damaged cells, and immune complexes [45]. The complement cascade is thought to play its traditional role of opsonizing and removing the cellular debris from widespread RGC death. As noted, the DBA/2J mice that did not develop glaucoma (DBA/2J_NG) showed similar C4a levels at 4, 10, and 14 months of age, and therefore this complement protein remains almost constant in the control group. However, a significant decrease during ageing was observed in DBA/2J mice developing glaucoma (DBA/2J_G^4^ vs. DBA/2J_G^10^ or DBA/2J_G^4^ vs. DBA/2J_G^14^, 0.7 fold-change), which may be related with the disease onset. Interestingly, C4a down-regulation has been also observed at a systemic level in humans with glaucoma (POAG or PEXG, 0.8-fold) [14]. The C4a small fragment, which is part of the classical activation pathway of the complement, acts on specific receptors triggering inflammatory response and inducing smooth muscle contraction and vascular permeability [46]. Our observations may suggest an important role of C4a in the early stages of glaucoma that warrants further investigations.

Similarly, APOA4 levels significantly decreased in the glaucoma group DBA/2J_G at 14 months of age, when compared to pre-glaucomatous 4 month old mice (DBA/2J_G^4^ vs. DBA/2J_G^14^, 0.56-fold change). However, the control group of DBA/2J mice not developing glaucoma (DBA/2J_NG) also provided a decrease in the levels of the protein during ageing (0.65-fold change when comparing 4 vs. 14 months), although without statistical significance (*p*-value > 0.05), probably due to the low number of samples in the groups. Moreover, the absence of differences when comparing DBA/2J_G^14^ vs. DBA/2J_NG^14^ and DBA/2J_G^4^ vs. DBA/2J_NG^4^ may limit the relevance of our observations and invalidate the APOA4 as individual biomarker in the discrimination of glaucomatous and non-glaucomatous subjects. Nevertheless, the significant deregulation of APOA4 during the development of glaucoma in mice may have unknown implications related with systemic alterations of immune response. Conversely, in glaucomatous human patients we observed an overexpression of APOA4, both in POAG and PEXG, which is related with immune-inflammatory response [14]. It should be noted that previous proteomic analysis in tears of glaucoma patients suggested variation in APOA4 levels [47], and recently this protein has been found as a constituent of the pseudoexfoliative material [48].

Proteins CFH, TTR, and ITIH4 experimented a significant increase in glaucomatous DBA/2J mice (from 4 to 10 and 14 months), with remarkably lower levels for CFH and ITIH4 at 4 months when compared with “healthy subjects”, i.e., D2G mice. Those early-stage low basal levels may be related with pre-disposition to develop glaucoma or be an indicative of subsequent onset of the disease. CFH protein is essential in regulating the complement activation since it accelerates the decay of the alternative C3 convertase [49]. Besides, TTR and ITIH4 have been previously identified altered in the human aqueous humor of glaucoma patients [50,51,52]. While the TTR is responsible for the transport of thyroxin and retinol binding protein complex to various parts of the body [53], the ITIH4 is related to the acute phase response and therefore involved in immunity and inflammation [54].

It should be noted that since the amount of serum samples from DBA/2J_NG mice was scarce and insufficient to quantify all selected proteins, the serum levels of CFH and ITIH4 in DBA/2J_G have been compared with those of the D2G control strain, at the same age (Table 1). Nevertheless, unlike the DBA/2J, the D2G strain expresses the functional transmembrane glycoprotein NMB (GPNMB), so significant differences could exist in the innate immune responses of D2G and DBA/2J mice, which may affect the obtained results. Evidence of GPNMB anti-inflammatory function has been previously described. For example, overexpression of GPNMB in macrophages reduces the secretion of pro-inflammatory cytokines [55]. Moreover, GPNMB promotes the polarization of macrophages into an anti-inflammatory status resulting in the secretion of anti-inflammatory cytokines [56,57]. On the other hand, increasing GPNMB levels were reported in patients with Alzheimer’s disease, suggesting anti-inflammatory, regenerative, and neuroprotective roles [58]. Considering that the DBA/2J mice do not present the functional GPNMB protein, systemic inflammatory status could be expected. However, while the DBA/2J mice presented lower levels of CFH and ITIH4 at 4 months, the same animals had normal levels at 10 and 14 months, when compared with D2G at the same age. Thereby, the observed systemic effects not only depend on the presence of the functional protein, although the differences obtained for CFH and ITH4 in the comparison of both groups (DBA/2J_G vs. D2G) must be taken very cautiously.

To improve clinical testing of glaucoma, specific biomarkers are needed for early diagnosis and prediction of its prognosis [59,60]. In recent years, great efforts in the field of glaucoma biomarkers discovery have been carried out, but there still remains the clinical validation phase to calculate the biomarker sensitivity and specificity in large and heterogeneous populations and, most importantly, their follow-up along the pre- and post-glaucomatous transitions in humans. In this work, the animal model DBA/2J was permitted to determine the alterations of candidate biomarkers throughout the different stages of the disease. Even more, using the ELISA data of the 10 serum proteins, a five-protein panel, consisting of C4a, CFH, FCN3, APOA4, and TTR, was obtained and used to generate machine-learning models (see Table 2). It should be noted that the percentage correct assignment when comparing 4-month vs. 10-month old mice developing glaucoma, i.e., the classification accuracy, predicts the transition to glaucoma in the 78% of cases. Similarly, the five-protein panel correctly classified the 89% of cases with advanced glaucoma. However, it must be stressed that the limited number of samples used for predicting glaucoma transitions may affect the discriminating power of the studied panel using the proposed algorithms. As expected, the PPI network of these five proteins shows that they are involved in immunological pathways, among which the activation of the complement system stands out. These results suggest that systemic alterations of proteins involved in immunological responses could be associated with the development of glaucoma in DBA/2J mice.

According to the literature, studies addressing systemic molecular changes during glaucoma progression in DBA/2J mice have not been reported. However, several limitations must be highlighted in the current study, including the small number of samples, specifically in the DBA/2J_NG control group, which has led to the use of the D2G strain as a control in some of the analysis. Although trained personnel performed blood collection, it was not always feasible to obtain enough serum from each animal to determine the concentration of the studied proteins. Therefore, the obtained preliminary results must be carefully considered, despite the significant differences obtained in some proteins, being necessary their validation in additional studies by increasing the number of animals and using DBA/2J_NG mice samples as controls in all cases. Moreover, future studies may include the analysis of the glaucomatous stage of DBA/2J mice at 10 months old or younger to classify those mice with initial glaucoma and search for possible changes in the levels of serum proteins when compared to pre-glaucomatous stages. On the other hand, the lack of the complement component C5 in both DBA/2J and D2G mice could hamper extrapolation of the results from mice to humans, with intact complement cascade. Furthermore, although in the past ELISA assays have been commonly used for the quantitative determination of one protein at the time, an alternative approach to quantitate candidate biomarkers in a larger number of samples without requiring antibodies is mandatory to warrant further validation in readily available biological fluids of glaucoma subjects. All this together with the analysis of additional candidate biomarkers related with immune response and complement system and the integration of data from omics studies (transcriptomics and metabolomics) may contribute to shed light into the pathophysiology of glaucoma and propose combined biomarkers for the early diagnostics of the disease.

## 5. Conclusions

In summary, our data suggest that disease development in DBA/2J mice is associated with important molecular changes in immune response and complement system proteins. The significant decrease of C4a in DBA/2J mice developing glaucoma could suggest a role of this protein in the disease onset. Moreover, the five-protein panel, consisting of C4a, CFH, FCN3, APOA4, and TTR, predicted the transition to glaucoma in 78% of cases and correctly classified 89% of cases with advanced glaucoma in this animal model. Although these observations need to be further investigated, this study provides new insights in the utility of the glaucoma mouse model DBA/2J to identify/propose marker(s) of potential use for the early diagnosis of glaucoma at systemic level.

## Figures and Tables

**Table 1:** Concentrations of proteins (ng/μL) obtained by ELISA analysis in serum of glaucoma and control groups.

		**Glaucoma Group**			**Control Group**			
**Protein**	**Conc.**	**DBA/2J_G^4^**	**DBA/2J_G^10^**	**DBA/2J_G^14^**	**DBA/2J_NG^4^**	**DBA/2J_NG^10^**		**DBA/2J_NG^14^**
**APOA1**	**n**	22 (14F/8M)	21 (13F/8M)	20 (12F/8M)	5 (4F/1M)	7 (6F/1M)		7 (6F/1M)
	**ng/µL**	764.74	716.27	735.00	695.39	651.12		662.65
	**SD**	197.97	174.58	203.06	246.05	120.66		109.21
**APOA4**	**n**	19 (11F/8M)	19 (11F/8M)	19 (11F/8M)	7 (6F/1M)	7 (5F/2M)		7 (4F/3M)
	**ng/µL**	22.26	17.88	12.38	24.94	26.64		16.46
	**SD**	4.82	5.84	1.87	3.20	4.32		3.00
**C3**	**n**	20 (13F/7M)	19 (13F/6M)	20 (13F/7M)	6 (5F/1M)	6 (5F/1M)		8 (6F/2M)
	**ng/µL**	137.06	120.7	140.88	161.06	112.26		155.27
	**SD**	47.82	19.26	46.24	44.71	21.51		47.24
**C4a**	**n**	22 (14F/8M)	20 (13F/7M)	20 (12F/8M)	4 (3F/1M)	7 (5F/2M)		7 (5F/2M)
	**ng/µL**	3.29	2.45	2.34	3.43	3.14		3.41
	**SD**	0.63	0.81	0.54	0.20	0.53		0.68
**TTR**	**n**	19 (12F/7M)	20 (13F/7M)	20 (13F/7M)	5 (4F/1M)	8 (6F/2M)		8 (6F/2M)
	**ng/µL**	22.94	28.59	27.53	24.58	22.36		26.45
	**SD**	3.76	3.90	7.80	4.59	8.83		6.44
		**Glaucoma Group**			**Control Group**			
**Protein**	**Conc.**	**DBA/2J_G^4^**	**DBA/2J_G^10^**	**DBA/2J_G^14^**	**D2G^4^**		**D2G^14^**	
**a1AT**	**n**	20 (12F/8M)	19 (11F/8M)	20 (12F/8M)	12 (11F/1M)		12 (11F/1M)	
	**ng/µL**	557.87	521.57	530.55	475.14		507.40	
	**SD**	227.17	186.89	214.91	161.21		81.90	
**APOL1**	**n**	20 (12F/8M)	19 (11F/8M)	20 (12F/8M)	14 (11F/3M)		14 (11F/3M)	
	**ng/µL**	2.51	2.19	2.25	2.09		2.53	
	**SD**	0.41	0.50	0.48	0.39		0.41	
**CFH**	**n**	18 (11F/7M)	19 (11F/8M)	20 (12F/8M)	14 (11F/3M)		14 (11F/3M)	
	**ng/µL**	68.31	89.67	89.67	84.04		88.02	
	**SD**	10.36	9.38	12.87	10.46		9.04	
**ITIH4**	**n**	18 (11F/7M)	19 (11F/8M)	19 (13F/6M)	14 (11F/3M)		14 (11F/3M)	
	**ng/µL**	73.41	112.99	112.04	98.98		126.71	
	**SD**	23.73	27.56	26.68	31.27		25.39	
**FCN3**	**n**	20 (12F/8M)	18 (10F/8M)	19 (11F/8M)	14 (11F/3M)		14 (11F/3M)	
	**ng/µL**	442.00	530.20	460.10	352.71		479.07	
	**SD**	177.63	225.59	178.87	134.78		210.25	

n, number of serum samples; SD, standard deviation; F, females; M, males.

**Table 2:** Best classifying machine-learning models using the protein panel C4a, CFH, FCN3, APOA4, and TTR.

Comparison	Method	CA	Sens.	Spec.	AUC	Prec.
**DBA/2J_G^4^ vs. DBA/2J_G^10^ vs. DBA/2J_G^14^**	Random Forest	0.62	0.74	0.90	0.84	0.80
**DBA/2J_G^4^ vs. DBA/2J_G^14^**	Naive Bayes	0.89	0.93	0.85	0.96	0.86
**DBA/2J_G^4^ vs. DBA/2J_G^10^**	Naive Bayes	0.78	0.80	0.77	0.88	0.76
**DBA/2J_G^10^ vs. DBA/2J_G^14^**	Random Forest	0.65	0.43	0.85	0.68	0.72

CA, correct assignment; Sens., sensitivity; Spec., specificity; AUC, area under the curve; Prec., precision.

## Supplementary Materials

The following are available online at https://www.mdpi.com/2075-4418/10/6/425/s1, Table S1: Analytical parameters of individual ELISA analysis.

## Abbreviations

a1AT	alpha 1-antitrypsin
APOA1	apolipoprotein A1
APOA4	apolipoprotein A4
APOL1	apolipoprotein L1
AUC	area under the curve
C3	complement C3
C4a	complement C4a
C5	complement component 5
CA	correct assignment
CFH	complement factor H
D2G	DBA/2J-*Gpnmb*+/SjJ mice strain without signs of glaucoma
DBA/2J_G	DBA/2J mice that developed glaucoma with the age
DBA/2J_NG	DBA/2J mice that did not develop glaucoma
FCN3	ficolin-3
GPNMB	transmembrane glycoprotein NMB
IOP	intraocular pressure
IPD	iris pigment dispersion
ISA	iris stromal atrophy
ITIH4	inter-alpha-trypsin inhibitor heavy chain H4
MOD	nerves with moderate damage
NOE	nerves with neither or early damage
PEXG	pseudoexfoliation glaucoma
POAG	Primary open-angle glaucoma
PPD	paraphenylenediamine
PPI	protein–protein interaction
Prec.	precision
RGC	retinal ganglion cell
Sens.	sensitivity
SEV	nerves with severe glaucoma
Spec.	specificity
TTR	transthyretin
